# Supplementary material for: Spatiotemporal isolation of attosecond soft X-ray pulses in the water window
Source: Nat Commun. 2015 Mar 19;6:6611. doi: 10.1038/ncomms7611 (PMC4382990; doi:10.1038/ncomms7611)
Supplement: Supplementary Information — Supplementary Figures 1-3 and Supplementary Note 1 [file ncomms7611-s1.pdf]

Supplementary Figures

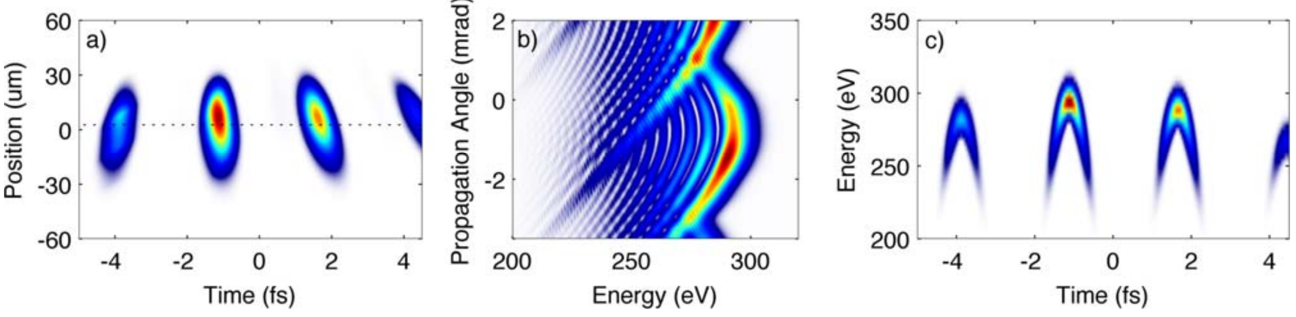

Supplementary Figure 1: Simulation results considering the full single atom response. (a) Generated spatio-temporal profile in focus. (b) Spatio-spectral profile in the far-field, for an angular range of 5.5 mrad. (c) Spectrogram of the pulse, calculated for the electric field along the dotted line in Supplementary Fig. 1(a).

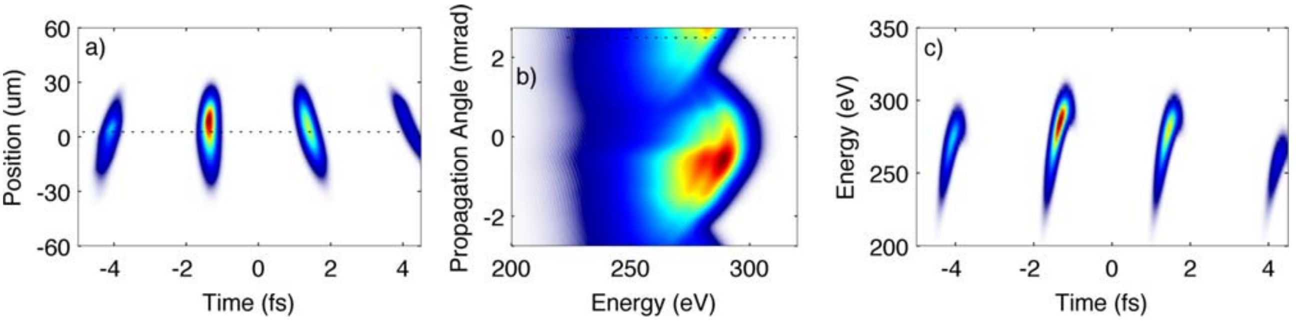

Supplementary Figure 2: Simulation results considering only short trajectories. (a) Generated spatio-temporal profile in focus. (b) Spatio-spectral profile in the far field, for an angular range of 5.5 mrad. (c) Spectrogram of the pulse, calculated for the electric field along the dotted line in Supplementary Fig. 2(a).

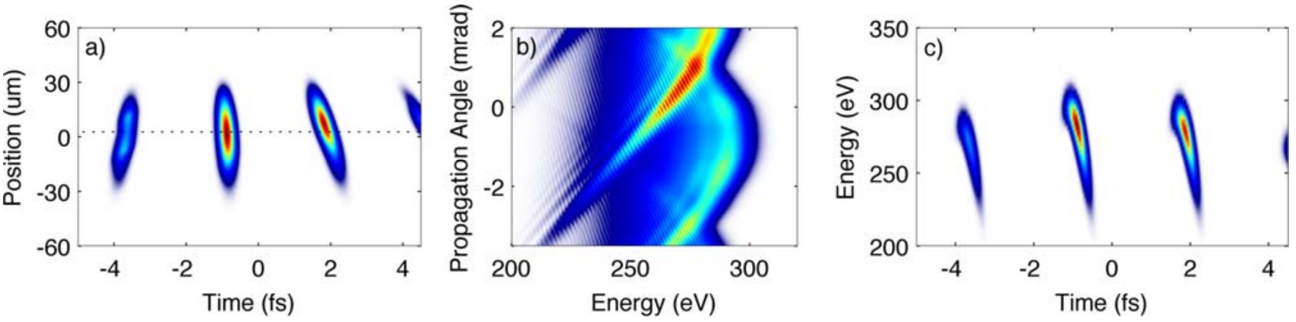

Supplementary Figure 3: Simulation results considering only long trajectories. (a) Generated spatio-temporal profile in focus. (b) Spatio-spectral profile in the far field, for an angular range of 5.5 mrad. (c) Spectrogram of the pulse, calculated for the electric field along the dotted line in Supplementary Fig. 3(a).

Supplementary Notes

Supplementary Note 1

Single-atom Response Calculation with Wavefront Rotation.

For the simulation results of Figs. 4 and 5 of the paper we model high harmonic generation by calculating the single-atom dipole moments, analog to Ref. (21). We assumed Gaussian profiles both in space and time for the driving laser pulse, with a width and wave-front rotation matching the measured spatio-spectral profile in Fig 1(a). The amplitude and phase of the single-atom dipole moments are calculated with the same procedure as with the 3D propagation code described in the methods, except in a (t,y) grid. The resulting $E_{Xray}(\omega,y)$ is then multiplied by the transmission of a 100 nm Aluminum filter and the transmission of neon gas after the target. In order to simulate the observed far field pattern E_xray(omega, angle) one can calculate the $E_{XRay}(\omega,k_T)$ by means of a Fourier transform and then transform from transverse momentum into propagation angle. Similar to (21), we restrict the sets of trajectories with a suitable temporal window to the calculated single atom dipole calculation.

Short vs Long trajectories with WFR.

We compare simulation results for the different trajectories. In Supplementary Fig. 1 we have represented the equivalent to Fig. 4 but considering the full single atom dipole response. By examining Supplementary Fig. 1(b) one can find that the presence of both families of trajectories leads to a strong spatio-spectral interference, with varying period. When either set of trajectories is removed we get the results in Supplementary Fig. 2 (short only) and Supplementary Fig. 3 (long only). We find, in accordance with Ref. (34), the long trajectories lead to more divergent far field beamlet profiles with dimensions much larger than the experimentally observed ones. When considering only short trajectories, the smaller divergence leads to a beam size matching the experimental observations. This suggests that the experimental conditions were optimised to favour the short trajectories only.
